# Structural and Immunodiagnostic Characterization of Synthetic Antigen B Subunits From *Echinococcus granulosus* and Their Evaluation as Target Antigens for Cyst Viability Assessment

**DOI:** 10.1093/cid/cix1006

**Published:** 2017-11-15

**Authors:** Daniela Pagnozzi, Francesca Tamarozzi, Anna Maria Roggio, Vittorio Tedde, Maria Filippa Addis, Salvatore Pisanu, Gabriella Masu, Cinzia Santucciu, Ambra Vola, Adriano Casulli, Giovanna Masala, Enrico Brunetti, Sergio Uzzau

**Affiliations:** 1 Porto Conte Ricerche, Science and Technology Park of Sardinia, Tramariglio, Alghero (Sassari), Italy; 2 Department of Clinical, Surgical, Diagnostic and Pediatric Sciences, University of Pavia, Italy; 3 WHO Collaborating Centre for the Clinical Management of Cystic Echinococcosis, Pavia, Italy; 4 National Reference Laboratory of Cystic Echinococcosis, Istituto zooprofilattico sperimentale della Sardegna “G. Pegreffi”, Sassari, Italy; 5 Division of Infectious and Tropical Diseases, IRCCS San Matteo Hospital Foundation, Pavia, Italy; 6 WHO Collaborating Centre for the Epidemiology, Detection and Control of Cystic and Alveolar Echinococcosis, Department of Infectious Diseases, Istituto Superiore di Sanità, Rome, Italy; 7 European Union Reference Laboratory for Parasites (EURLP), Department of Infectious Diseases, Istituto Superiore di Sanità, Rome, Italy; 8 Department of Biomedical Sciences, University of Sassari, Italy

**Keywords:** Cystic echinococcosis, AgB, serodiagnosis, structural and immunological characterization, cyst viability

## Abstract

**Background:**

Several tools have been proposed for serodiagnosis of cystic echinococcosis (CE), but none seems promising for cyst viability assessment. Antigens with stage-specific diagnostic value have been described, but few studies with well-characterized antigens and human serum samples have been performed. Antigen B (AgB) proteoforms hold promise as markers of viability, due to their differential stage-related expression and immunoreactivity.

**Methods:**

Four AgB subunits (AgB1, AgB2, AgB3, AgB4) were synthesized and structurally characterized. Based on the preliminary evaluation of the subunits by western immunoblotting and enzyme-linked immunosorbent assay (ELISA), AgB1 and AgB2 were further tested in two ELISA setups and extensively validated on 422 human serum samples.

**Results:**

All subunits showed a high degree of spontaneous oligomerization. Interacting residues within oligomers were identified, showing that both the N-terminal and C-terminal of each subunit are involved in homo-oligomer contact interfaces. No hetero-oligomer was identified. AgB1 and AgB2 ELISAs revealed different sensitivities relative to cyst stage. Of note, besides high specificity (97.2%), AgB1 revealed a higher sensitivity for active-transitional cysts (100% for CE1, 77.8% for CE2, 81.5% for CE3a, and 86.3% for CE3b) than for inactive cysts (41.7% for CE4 and 11.1% for CE5) and postsurgical patients (44%). Interestingly, 19 of 20 patients with spontaneously inactive cysts and 6 of 9 treated with albendazole >5 years earlier were negative on the AgB1 assay.

**Conclusions:**

The structural characterization of subunits provides insights into the synthetic antigen conformation. The stage-related sensitivity of synthetic AgB1 holds promise as part of a multiantigen setting and deserves further longitudinal evaluation as marker of cyst viability.

## Teaser

The available cystic echinococcosis immunodiagnostic tools cannot assess cyst viability. Here, synthetic antigen B (AgB) subunits were structurally and immunologically characterized. They all showed propensity to self-oligomerize, similarly to native AgB, and AgB1 held promise as a marker of viability.

Cystic echinococcosis (CE) is a neglected zoonotic disease caused by the larval stage of the parasite *Echinococcus granulosus* sensu lato and represents a worldwide public health and economic issue. The life cycle typically includes sheep, goats, swine, and cattle among others as intermediate hosts, and canids as definitive hosts, and the parasite is particularly widespread in livestock breeding areas [1]. Humans may act as accidental intermediate hosts by ingestion of parasite eggs in contaminated material. After infection, fluid-filled cysts develop that can localize in different organs, mostly liver and lungs, and may remain asymptomatic for years.

Although imaging, especially ultrasonography (US) for abdominal localization, is the most reliable tool for diagnosis of CE, serology is a useful complementary tool. However, the performances of current serological tests are unsatisfactory [2, 3].

The World Health Organization Informal Working Group on Echinococcosis international classification of US images classifies CE cysts in 6 stages [4, 5], which largely reflect the biological viability of cysts: CE1, CE2, CE3b (CE1–2, active cysts, CE3b, transitional cyst; all biologically viable), CE3a (transitional cyst; variable viability), and CE4 and CE5 (inactive cysts; biologically nonviable) [6]. However, the biological nonviability of inactive cysts cannot be predicted based on US appearance. In particular, CE4 cysts (inactive cysts with solid appearance at US) are in the vast majority of cases nonviable when this stage is reached spontaneously [7–9], and a variable proportion of cases reactivate if CE4 has been reached after medical treatment of an active stage, indicating biological viability [10, 11]. Hence the need for years-long follow-up with US to detect cyst reactivation after treatment, as currently available serological tests are not useful for this purpose [12].

Many investigations evaluated the diagnostic performances of synthetic, native, or recombinant purified antigens of *E. granulosus*. Antigen 5 (Ag5) and antigen B (AgB) are by far the most exploited, owing to their abundance and immunogenic properties [13, 14]. AgB is an oligomeric thermostable lipoprotein encoded by 5 genes (AgB1–AgB5), composed of multimers of 8-kDa subunits [15], mainly secreted by the germinal layer [16], and carrying a large amount of lipids of host origin, which account for almost 50% of its mass. Several studies have investigated the reactivity of AgB1 and AgB2, the most abundant subunits in the native complex [17]. AgB has also been structurally investigated [18–22], revealing an α-helix–rich configuration, with a high propensity to oligomerize in both the native and recombinant forms.

Given their abundance and immunogenicity, both Ag5 and AgB have been often proposed as targets in serological assays. However, mixed results have been obtained for these antigens in terms of sensitivity and specificity [13, 14], probably owing to the use of different experimental conditions, different panels of serum samples, limited number of samples, poor interlaboratory reproducibility of antigen preparation, and lack of information about the antigen structure, that can heavily affect its immunoreactivity [2, 23, 24].

To this aim, we have previously highlighted the critical role of a high-quality purification of native Ag5 for developing highly specific serological assays [25, 26]. Different protein targets, including Ag5 and high-quality products of the AgB gene variants, might be combined in a multiantigen test for enabling a more reliable serological diagnosis and providing useful indications on cyst viability. However, a rapid, high-quality, and high-yielding purification of native AgB variants represents an unmet challenge, and synthetic products would serve as an excellent alternative. However, the structural features of a given synthetic peptide deserve to be investigated to deepen the knowledge on the possible relationship between the in vitro obtained structures and their immunoreactivity in novel serological assays.

In this study, therefore, we characterized the structural features and evaluated the immunoreactivity of four synthetic proteins, corresponding to the subunits AgB1–AgB4, the main constituents of the native AgB complex [18]. We provide evidence of their propensity for oligomerization and report detailed information on interacting residues, as well as the first putative description of AgB homo-oligomers arrangement. Building on these well-characterized synthetic antigens, we also extended the evaluation of the different immunoreactivities exhibited by AgB proteoforms carried out by Ahn and coworkers [17], to cyst stages. To this end, serum samples from a cohort of patients and healthy controls were tested against synthetic AgB1 and AgB2 in two enzyme-linked immunosorbent assay (ELISA) setups. The same serum samples were also assayed with the Ag5 setup [26]. We observed different sensitivities depending on the AgB subunit and cyst stage, and a good association between AgB1 negative ELISA results and the presence of untreated (ie, spontaneously inactivated) inactive cysts. The comparison of AgB and Ag5 ELISA results opens interesting perspectives on the applicability of a multiantigen test for the assessment of cyst viability and follow-up of patients.

## METHODS

A detailed description of methods is provided in Supplementary File S1.

### Synthesis and Purification of AgB Subunits

AgB1, AgB2, AgB3, and AgB4 sequences (7.6, 8.2, 7.7, and 8.2 kDa, respectively) were selected from the UniProtKB database. Proteins were synthesized, purified, and verified using mass spectrometry (MS) [27, 28].

### Size-Exclusion Chromatography

Size-exclusion chromatography (SEC) was performed in 50 mM sodium phosphate, 150 mM NaCl, pH 7.4, on a AKTA Explorer 10 system. Elution profiles were recorded, based on the UV absorption at 220 and 280 nm.

### Chemical Cross-Linking Experiments

Each AgB subunit or its equimolar mixture was treated with a 100-fold molar excess of 1-ethyl-3-(3-dimethylaminopropyl)carbodiimide (EDC) cross-linking reagent; reactions were stopped, and samples were separated by sodium dodecyl sulfate–polyacrylamide gel electrophoresis (SDS-PAGE) [29]. Selected protein bands were in situ digested [30], and analyzed using liquid chromatography–tandem MS performed with a Q Exactive mass spectrometer [31] interfaced with an UltiMate 3000 RSLCnano liquid chromatography system. Cross-linked peptides were identified using StavroX software (version 3.3.0.1) [32].

### Western Immunoblotting

Western immunoblotting was performed as described elsewhere [25]. Membranes were incubated with 1:200 Working Standard Anti-Echinococcus Serum, Human (WSH serum).

### Serum Samples

A total of 422 blood serum samples were collected, including 148 from patients with CE cysts, 25 from patients who had previously been treated surgically for CE and in follow-up at the time of serum collection (after surgery), and 249 from healthy subjects. Table 1 summarizes the number of patients available for each group and the corresponding cyst location.

**Table 1.** Patient Grouping According to Clinical Characteristics and Location of the Cysts With the Stage Likely Most Influencing Their Serological Status

Group	Patients, No.				Patients, No. (%)	
	Total	No Albendazole Treatment	Albendazole <5 y Earlier^a^	Albendazole >5 y Earlier^b^	Hepatic Cysts	Extrahepatic Cysts
CE1	7	2	5	0	7 (100.0)	0
CE2	9	2	7	0	9 (100)	0
CE3a	27	7	20	0	26 (96.3)	1 (3.7)
CE3b	51	14	37	0	44 (86.3)	7 (13.7)
CE4	36	8	23	5	34 (94.4)	2 (5.6)
CE5	18	12	2	4	16 (88.9)	2 (11.1)
Postsurgical	25	0	25	0	21 (84.0)	4 (16.0)
Total	173	45	119	9	157 (90.8)	16 (9.2)

a Albendazole intake ended <5 years before collection of serum samples for this study.

b Albendazole intake ended >5 years before collection of serum samples for this study.

### ELISA

ELISA was performed as described elsewhere [26]. Serum samples were added at 1:200 dilutions to microplates coated with 100 µL per well of antigen solutions in phosphate-buffered saline. To compare results obtained from different plates, we calculated a sample ratio .

### Statistical Analysis

Data analysis was performed with MedCalc statistical software, version 15.2.2. Receiver operating characteristic (ROC) curves, McNemar test, and Kruskal-Wallis test were applied to data sets, as reported elsewhere [26, 33–36].

## RESULTS

### Structural Characterization of AgB Subunits

**Figure 1.**
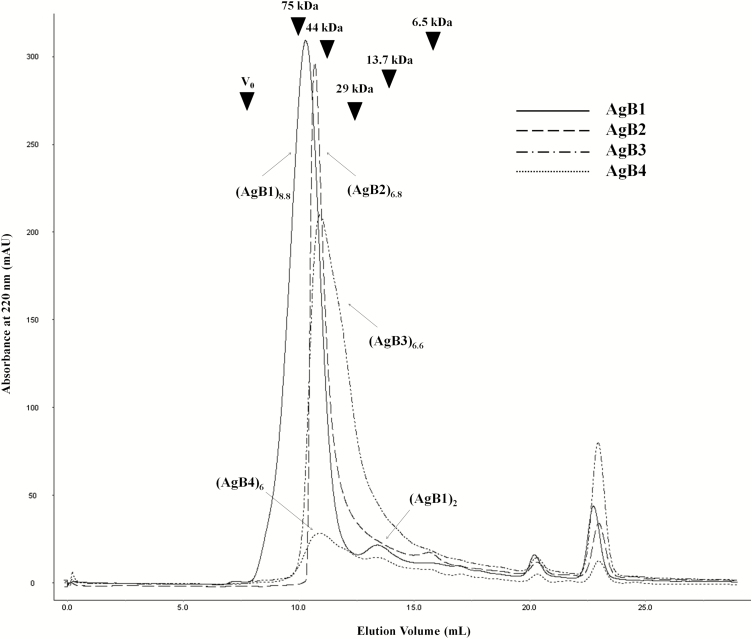
Size-exclusion chromatography of the synthetic antigen B (AgB) subunits. The chromatogram overlay of AgB1, AgB2, AgB3, and AgB4 shows their similar propensity to oligomerize. (AgBm)_*n*_ indicates the *n* units forming the AgBm oligomer. Size calibration marks, obtained from runs of standard proteins aprotinin (6.5 kDa), ribonuclease A (13.7 kDa), carbonic anhydrase (29 kDa), ovalbumin (44 kDa), conalbumin (75 kDa) and blue dextran 2000 (as void volume *V*_0_) are indicated by arrowheads in the chromatogram.

The oligomeric state of AgB1–AgB4 synthetic subunits was investigated by SEC and cross-linking experiments. SEC was performed on the four subunits to study their quaternary structure in phosphate buffer (Figure 1). AgB1 chromatography demonstrated a single main peak with an elution volume corresponding to a molecular weight of about 66 kDa, in agreement with an octamer/nonamer (8.8 units) and a very small peak at the dimer’s elution volume (17 kDa). For AgB2 and AgB3, a single peak was observed (about 56 and 51 kDa, respectively), both corresponding to esameric/eptameric structures (6.8 and 6.6 units, respectively). AgB4 exhibited only a very small peak, probably owing to solubilization issues or a high propensity to aggregate, at an apparent molecular weight of about 51 kDa (esamer).

**Figure 2.**
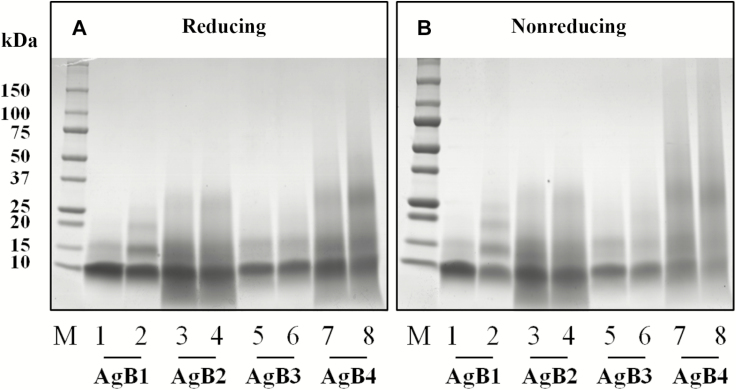
Sodium dodecyl sulfate–polyacrylamide gel electrophoresis of synthetic antigen B (AgB) subunits and their cross-linking products under reducing (*A*) and nonreducing conditions (*B*). Lanes 1, 3, 5 and 7 correspond to the individual subunits without the addition of 1-ethyl-3-(3-dimethylaminopropyl)carbodiimide (EDC) reagent. Lanes 2, 4, 6, and 8 correspond to the individual subunits after incubation with EDC reagent. Abbreviation: M, molecular weight markers.

Cross-linking experiments with EDC were performed to covalently stabilize the quaternary structure reached by each subunit or an equimolar mixture of the four proteins, to allow self-assembling in heteromeric complexes. Products were separated by SDS-PAGE in reducing and nonreducing conditions (Figure 2A and 2B for single subunits and Supplementary Figure S1A–S1B for the mixture, respectively). AgB1 oligomers clearly showed the formation of covalent bonds among subunits on the addition of EDC, leading to the appearance of bands putatively corresponding to dimers, trimers, and tetramers (Figure 2A and 2B, lane 2). Although for AgB2, AgB3, AgB4, and the mixture the effect of cross-linking was less evident, the gel bands putatively containing their dimers, trimers, tetramers, and, for the mixture, high-molecular-weight species, were also excised and analyzed by MS; the monomeric bands were analyzed as a control, to deplete signals deriving from internal cross-links.

**Figure 3.**
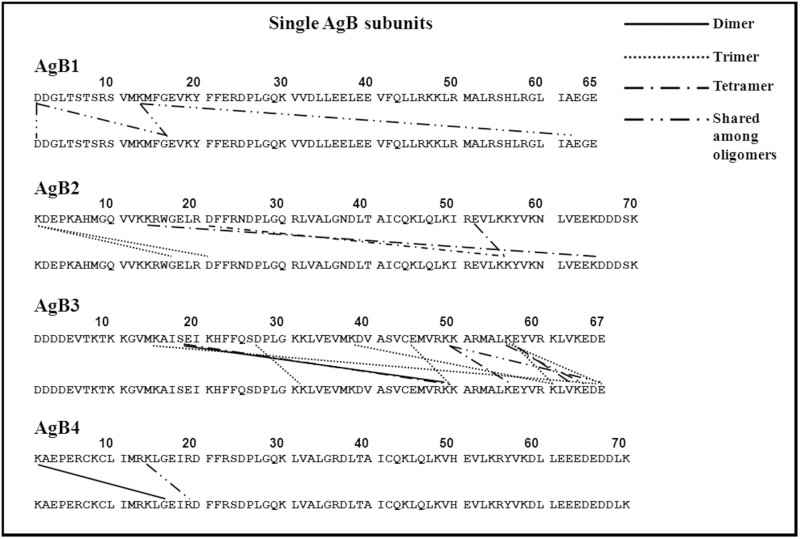
Interacting residues from mass spectrometric analysis of protein bands after incubation of single subunits with 1-ethyl-3-(3-dimethylamino propyl)-carbodiimide. Abbreviation: AgB, antigen B.

After careful attribution of all the interacting residues also found in the monomeric bands, StavroX software analysis, and manual check of the spectra, a series of cross-linked peptides was detected. Results are summarized in Figure 3, Supplementary Figure S2 and Supplementary File S2; the tandem MS spectrum of a cross-linked peptide is shown, as an example, in Supplementary Figure S3.

All the evaluated combinations provided information about cross-linked residues, revealing interactions involving both N- and C-terminal regions. The distribution of interacting residues was quite similar among the different subunits, both when they underwent cross-linking by themselves and in the mixture. No hetero-oligomer was identified, suggesting that, at least in our experimental conditions, subunits rearrange, also in an equimolar mixture, as homo-oligomers.

### Immunoreactivity of AgB Subunits and Oligomers

**Figure 4.**
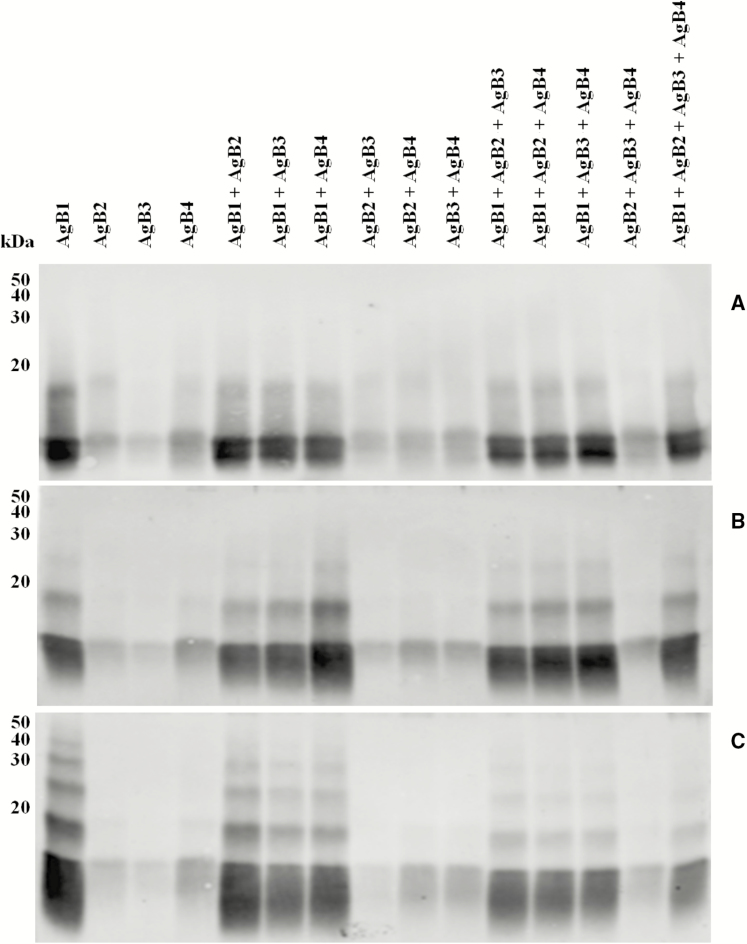
Western immublotting of synthetic antigen B (AgB) subunits (*A*) and their cross-linking products after incubation with a 10-fold (*B*) or a 100-fold (*C*) excess of 1-ethyl-3-(3-dimethylamino propyl)-carbodiimide under reducing conditions, as individual components, or as a mixture of the four subunits.

The immunoreactivity of AgB1, AgB2, AgB3, and AgB4 subunits, their combinations, and the corresponding cross-linked products were evaluated by western immunoblotting with WSH serum. Results are shown in Figure 4. Antigens were tested either as single AgB subunits or as an equimolar mixture. AgB1 showed the highest reactivity, followed by AgB4, AgB2, and AgB3, and the same trend was observed when the subunits were mixed. Noncovalent dimers (Figure 4A) were visible in the absence of EDC, highlighting the stability of their quaternary structure also in denaturing conditions. A 10-fold excess of cross-linker influenced AgB1, for which a trimer was slightly evident (Figure 4B). A 100-fold excess of EDC, though not showing AgB2, AgB3, and AgB4 reactive oligomers, resulted in the appearance of the AgB1 tetramer and pentamer, producing the classic ladderlike pattern of native AgB (Figure 4C and Supplementary Figure S4).

To assess the immunoreactivity of single AgB subunits and of different equimolar assortments also in non denaturing conditions, 25 ng/well, 33 ng/well, 50 ng/well, and 100 ng/well of each protein solution were tested by ELISA, with the WSH serum.

**Figure 5.**
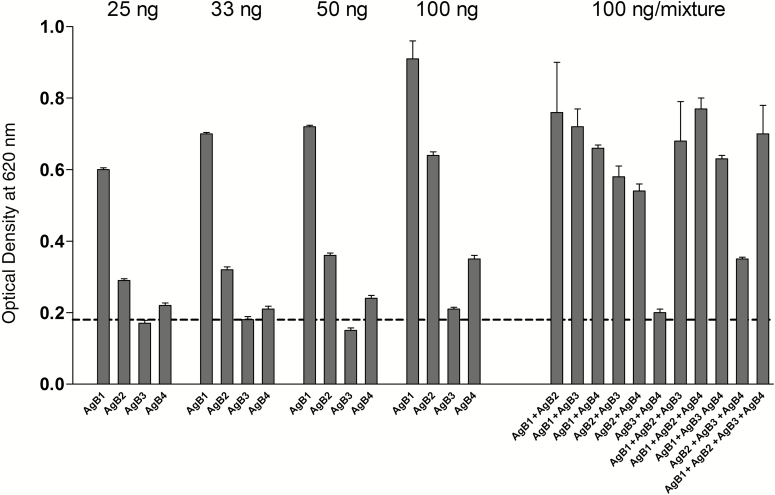
Enzyme-linked immunosorbent assay absorbance values using the synthetic subunits as antigens. Different amounts of each synthetic subunit (25, 33, 50 and 100 ng) and their equimolar combination (100 ng of total mixture) were evaluated against the Working Standard Anti-Echinococcus Human Serum. Dashed line indicates absorbance value obtained in the same conditions, on an uncoated well. Bars indicate standard deviations from the mean.

The absorbance values revealed different immunoreactivities of the four subunits, according to the following trend: AgB1 > AgB2 > AgB4 > AgB3 (Figure 5). No increase was observed when AgB1 was mixed to any other subunit, compared with the same amount (100 ng) of AgB1 individually coated, whereas the other combinations provided higher or lower absorbance values, depending on the antigens combined.

### Validation of AgB1 and AgB2 ELISA Setups With a Large Collection of Ser Samples

**Figure 6.**
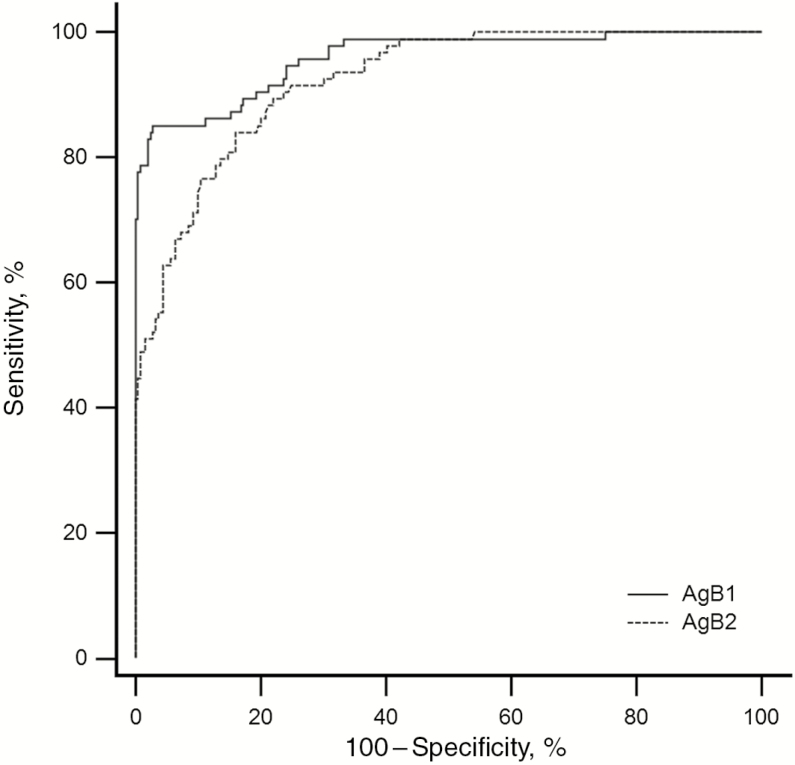
Receiver operating characteristic (ROC) curve overlay of the antigen B1 (AgB1) and antigen B2 (AgB2) enzyme-linked immunosorbent assay setups. Curves are generated by plotting sensitivity versus 100 − specificity.

AgB1 and AgB2, at 25 ng per well, were selected for individual validation experiments on a large panel of human serum samples. To characterize and validate their immunoreactivity, 422 serum samples from individuals harboring CE cysts in different stages were analyzed by ELISA. The optimal cutoff values were calculated based on ROC curves (Figure 6 and Table 2). The areas under the ROC curve were 0.959 and 0.923 for AgB1 and AgB2, respectively. At the best sample ratio cutoff value (0.103 and 0.058 for AgB1 and AgB2, respectively), AgB1 and AgB2 ELISAs showed very similar sensitivity toward patients with cysts in active-transitional stages (85.1 vs 84.0%; *P* = >.99) and postsurgical patients (44.0% vs 56.0%; *P* = .25) whereas differences were observed in patients with CE4 and CE5 cysts and healthy controls. 

**Table 2.** Statistical Parameters of AgB1 and AgB2 ELISA at the Best Cutoff Values

Antigen	Sensitivity, %	Specificity, %	AUC (SE) [95% CI]	Youden Index	Likelihood Ratio	
					Positive	Negative
AgB1	85.11	97.19	0.959 (0.012) [.932–.977]	0.8230	30.27	0.15
AgB2	84.04	83.94	0.923 (0.015) [.889–.949]	0.6798	5.23	0.19

Abbreviations: AgB1, antigen B1; AgB2, antigen B2; AUC, area under the (receiver operating characteristic) curve; CI, confidence interval; ELISA, enzyme-linked immunosorbent assay; SE, standard error.

**Figure 7.**
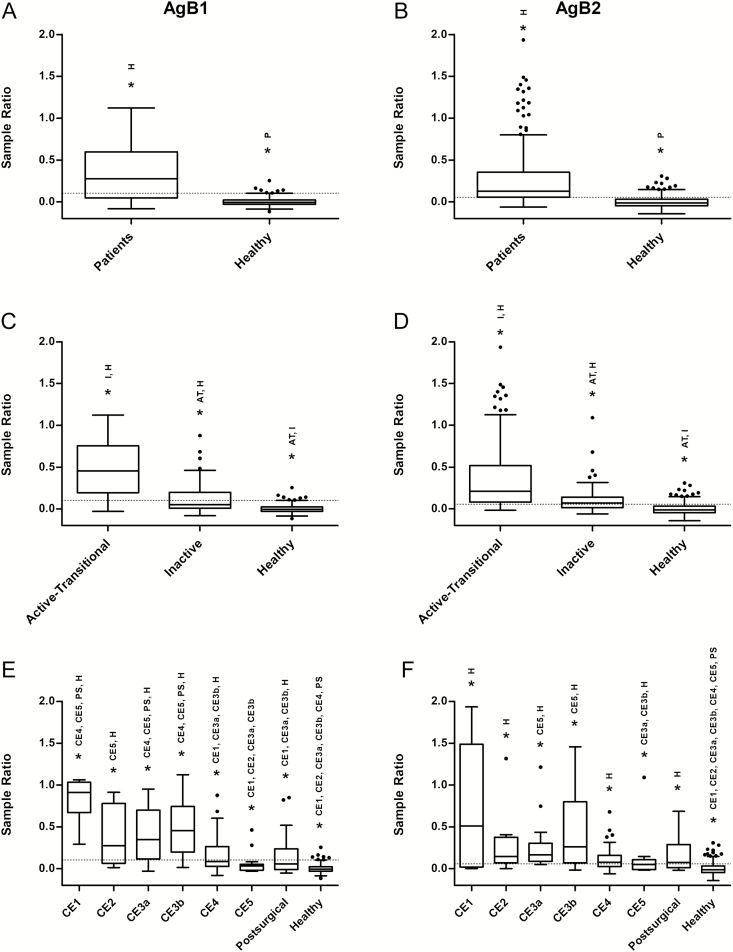
Box-and-whiskers plots of enzyme-linked immunosorbent assay results for antigen B1 (AgB1) (left panels) and antigen B2 (AgB2) (right panels) setups. Boxes indicate values falling within the 25th and 75th percentiles (interquartile range [IQR]). Central lines represent median values; whiskers, values falling within the ±1.5 IQR; single dots, values falling outside the whiskers; dashed lines, the best cutoff values. *Significantly different groups after Kruskal-Wallis test, or after Conover test, with Bonferroni correction, for multiple comparisons. According to this adjustment, to achieve statistical significance three *P* values have been considered: *P* < .05 for *A* and *B*, *P* < .02 for *C* and *D*, and *P* < .002 for panels *E* and *F*. Abbreviations: AT, active-transitional; H, healthy donors; I, inactive; P, patients; PS, postsurgical.

The positive rates in patients with CE4 and CE5 cysts were 41.7% and 11.1%, respectively, for AgB1 ELISA, and 69.4% and 38.9% for AgB2 ELISA. The specificity of AgB1 was higher than that of AgB2 (97.2% vs 83.9%), as tested on serum samples from healthy controls. Results are summarized in Table 3. Both ELISAs discriminated between patients and healthy controls (Figure 7A and 7B). Statistically significant differences were also found between patients with active-transitional versus inactive CE cysts (Figure 7C and 7D). Results obtained with serum samples according to cyst stages are shown in Figure 7E and 7F. AgB2 ELISA behaved differently from AgB1, because only patients with CE5 cysts and healthy donors showed significant differences from other subgroups.

**Table 3.** ELISA Results by Group

Group	Patients, No.	Positive by AgB1, No. (%)	Positive by AgB2, No. (%)	*P* Value
Active-transitional				
Total	94	80 (85.1)	79 (84.0)	>.99
CE1	7	7 (100.0)	5 (71.4)	.50
CE2	9	7 (77.8)	8 (88.9)	>.99
CE3a	27	22 (81.5)	25 (92.6)	.38
CE3b	51	44 (86.3)	41 (80.4)	.58
Inactive				
Total	54	17 (31.5)	32 (59.3)	<.001^a^
CE4	36	15 (41.7)	25 (69.4)	.01^a^
CE5	18	2 (11.1)	7 (38.9)	.06
Postsurgical	25	11 (44.0)	14 (56.0)	.25
Healthy controls	249	7 (2.8)	40 (16.1)	<.001^a^

Abbreviations: AgB1, antigen B1; AgB2, antigen B2; ELISA, enzyme-linked immunosorbent assay.

a Statistically significant difference (McNemar test).

**Figure 8.**
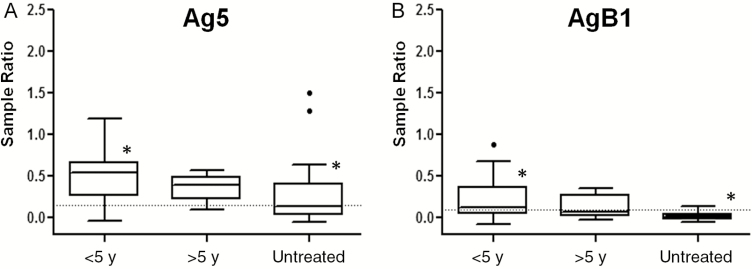
Box-and-whiskers plots of enzyme-linked immunosorbent assay results for antigen 5 (Ag5) (*A*) and antigen B1 (AgB1) (*B*) setups. Boxes represent values falling within the 25th and 75th percentiles (interquartile range [IQR]); central lines, median values; whiskers, values falling within ±1.5 IQR; single dots, values falling outside the whiskers; dashed lines, best cutoff values. *Significantly different after Kruskal-Wallis test, coupled to Conover test, with Bonferroni correction, for multiple comparisons (*P* < .02).

Finally, AgB1 ELISA results were compared with those for Ag5 [26], using the same 422 serum samples (Table 4). Specificity was higher for AgB1 than for Ag5, although this difference was not statistically significant. Furthermore, AgB1 was significantly less sensitive than Ag5 in detecting patients with cysts at any stage, but especially with inactive cysts. It is noteworthy that when patients with inactive cysts were further grouped into patients with spontaneously inactivated (untreated) cysts, or treated with albendazole either <5 or >5 years previously (and thus inactivated as the result of treatment), 10 of 20 untreated patients with inactive cysts, 23 of 25 treated with albendazole <5 years earlier, and 8 of 9 treated >5 years earlier, respectively, were positive to Ag5 (Figure 8A), compared with only 1 of 20, 13 of 25 and 3 of 9, respectively, to AgB1 (Figure 8B). Moreover, when the same grouping was evaluated for patients with active-transitional cysts, Ag5 results were positive in 22 of 25 untreated patients and 68 of 69 who ended albendazole intake <5 years before the collection of serum, whereas AgB1 results were positive in 21 of 25 and 58 of 69, respectively.

**Table 4.** Ag5 ELISA Serological Results and Comparison With AgB1 ELISA

Group	Patients, No.	Positive by Ag5, No. (%)	*P* Value
Active-transitional			
Total	94	90 (95.7)	.03^a^
CE1	7	7 (100.0)	NA
CE2	9	9 (100)	.50
CE3a	27	26 (96.3)	.22
CE3b	51	48 (94.1)	.34
Inactive			
Total	54	41 (75.9)	<.001^a^
CE4	36	31 (86.1)	<.001^a^
CE5	18	10 (55.6)	.008^a^
Postsurgical	25	19 (76.0)	.008^a^
Healthy controls	249	13 (5.2)	.21

Abbreviations: Ag5, antigen 5; AgB1, antigen B1; ELISA, enzyme-linked immunosorbent assay; NA, not applicable.

a Statistically significant difference (McNemar test).

## DISCUSSION

The search for a marker of cyst viability, to complement US staging, is one of the challenges in the field of clinical CE. The results of serodiagnostic tests are still unreliable from a clinical point of view. Several studies have been performed on small or medium/large panels of human serum samples, mostly involving a limited number of immunodominant proteins [13, 14]. The most extensively investigated and promising antigens are Ag5 and AgB, owing to their strong immunoreactivity and relatively high abundance in hydatid fluid. Both have been tested as native, recombinant, and synthetic antigens [13, 14], with conflicting results. A number of features make AgB a good candidate biomarker of CE development and progression, including its primary secretion by the germinal layer cells [16], its putative involvement in the parasite lipid metabolism and as potential ligand for monocyte and macrophage receptors [22, 37], the variation of its relative abundance, specific oligomer assortments, and/or exposition to the immune system at different CE stages [17]. 

From a structural point of view, some information on this antigen was provided by other authors, both on recombinant subunits and on the native AgB complex [18, 19, 38]. Understanding the protein conformation can help explain its immunoreactivity and provide information about the host-parasite interplay. Nevertheless, we are not aware of previous studies combining structural and immunological studies of this CE antigen. In the current study, we performed a detailed structural and immunodiagnostic investigation of the synthetic proteins constituting the four main subunits of the native AgB complex. According to the conformational studies, the ability of all the synthetic proteins to form oligomeric structures was shown for the first time. Our results suggest that the four subunits rearrange into highly organized structures, up to nonamers, and that interactions involve both the N- and the C-terminal regions, as the subunits would be organized in parallel and/or antiparallel bundles, maybe generating channels where lipids can be accommodated. 

As already suggested [18], we hypothesize that in vivo each subunit may act as a nucleation center whose propagation leads to high-order oligomers, also building on the presence of other compounds or external stimuli. Among the subunits, the most cross-linked was AgB3, confirming the previous observation about a more compact structure that could also be responsible for a lower immunoreactivity [18]. Furthermore, the proposed mechanism might explain the lack of conformational data obtained by both structural approaches on AgB4, whose low solubility may be due to its high propensity to oligomerize up to aggregation.

Although showing a similarity with AgB3 in the distribution of contact residues, AgB1 and AgB2 results indicated a higher flexibility, which may be responsible for their higher immunoreactivity. Further studies are warrant to confirm these hypotheses and elucidate their relevance, if any, in host-parasite interplay. Moreover, our experimental conditions were not able to stabilize any hetero-oligomer, but other experiments, exploring different setups and different approaches, would be required. Finally, AgB homo-oligomers partially survive SDS-PAGE (Figures 2 and 4), although to a lesser extent than native AgB. Our results agree with the previous observation that self-assembly does not depend on the presence of lipids, but in their absence oligomers may be smaller than in the native complex [38].

Having demonstrated the ability of synthetic subunits to mimic the native antigen, we tested their immunoreactivity. Serological assays based on synthetic subunits showed a clear immunoreactivity with serum samples from patients with CE, using both Western immunoblotting and ELISA, with differences between assays mostly involving AgB4. The AgB4 behavior might again be explained by the different assay conditions and the low solubility of AgB4 in phosphate buffer.

Both AgB1 and AgB2, the most reactive antigens, demonstrated a good sensitivity to active-transitional stages. On the contrary, AgB1 especially was poorly sensitive in patients with inactive cysts and after surgery. When we further investigated this noteworthy result, we found that AgB1 was very poorly sensitive in patients with spontaneously inactive cysts (who had never received albendazole therapy) and those with inactive cysts resulting from the treatment of active cysts (who had finished their last course of albendazole >5 years earlier and for which cysts is plausible to suppose biological nonviability). 

This finding suggests that AgB1 could be of use to discriminate between inactive cysts that are no longer biologically viable and those still biologically viable and therefore requiring a longer/closer follow-up with US to detect reactivation. However, these results need confirmation with a longitudinal study evaluating responses to AgB along the evolution of CE4 cysts over time. Our data also strongly support the use of both Ag5 and AgB1 in a multiantigen configuration, to provide both high sensitivity for diagnosis of CE, and information on cyst viability, to guide the follow-up approach. Antigen combinations should be further explored to identify the optimal setup conditions for developing a highly performing diagnostic test.

## Supplementary Data

Supplementary materials are available at *Clinical Infectious Diseases* online. Consisting of data provided by the authors to benefit the reader, the posted materials are not copyedited and are the sole responsibility of the authors, so questions or comments should be addressed to the corresponding author.

Supplementary Figure S1

Supplementary Figure S2

Supplementary Figure S3

Supplementary Figure S4

Supplementary File S1

Supplementary File S2

## Notes

***Acknowledgments.*** We thank the “HERACLES” extended network (http://www.Heracles-fp7.eu/) for the useful discussions among the members.

***Financial support.*** This work was supported by Regione Autonoma della Sardegna (Article 9 LR 20/2015).

***Potential conflicts of interest.*** All authors: No reported conflicts. All authors have submitted the ICMJE Form for Disclosure of Potential Conflicts of Interest. Conflicts that the editors consider relevant to the content of the manuscript have been disclosed.
