# Supplementary figures and images for: Structural and Immunodiagnostic Characterization of Synthetic Antigen B Subunits From Echinococcus granulosus and Their Evaluation as Target Antigens for Cyst Viability Assessment
Source: Clin Infect Dis. 2017 Nov 15;66(9):1342–51. doi: 10.1093/cid/cix1006 (PMC5905600; doi:10.1093/cid/cix1006)

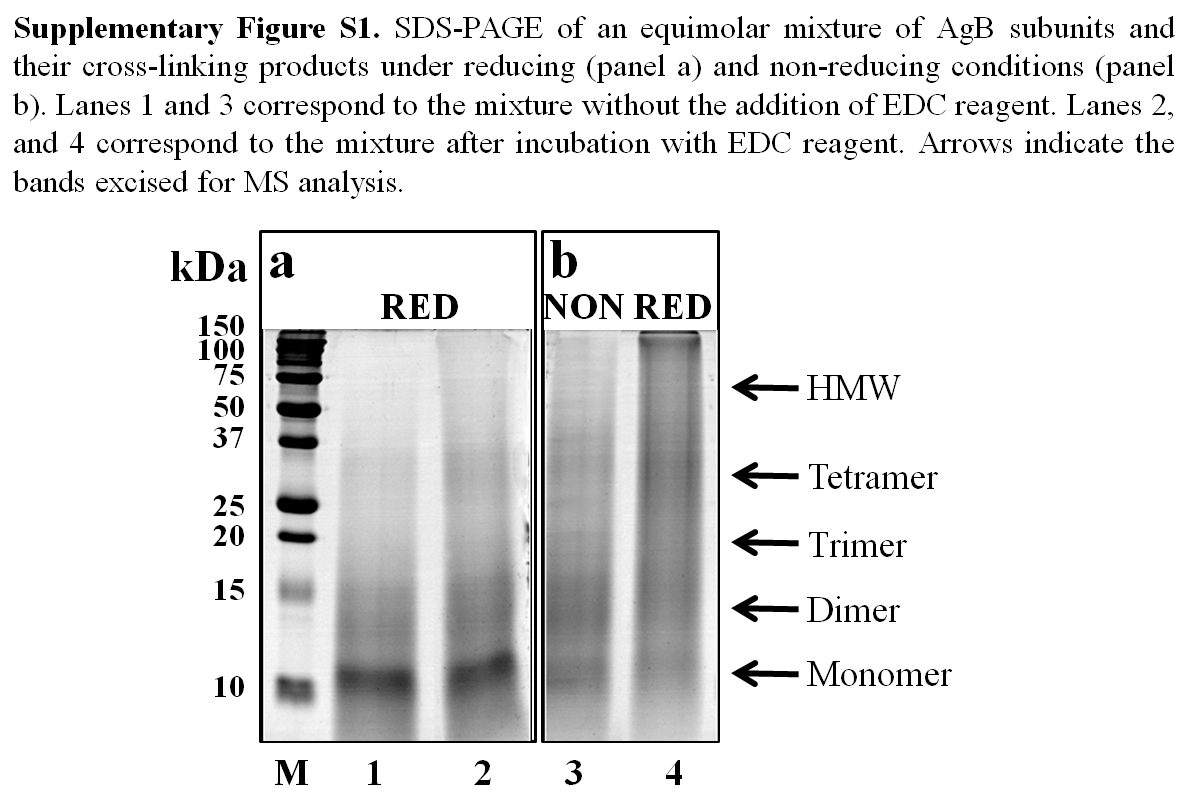

Supplement: Supplementary Figure S1 [file cix1006_suppl_supplementary_figure_s1.png]

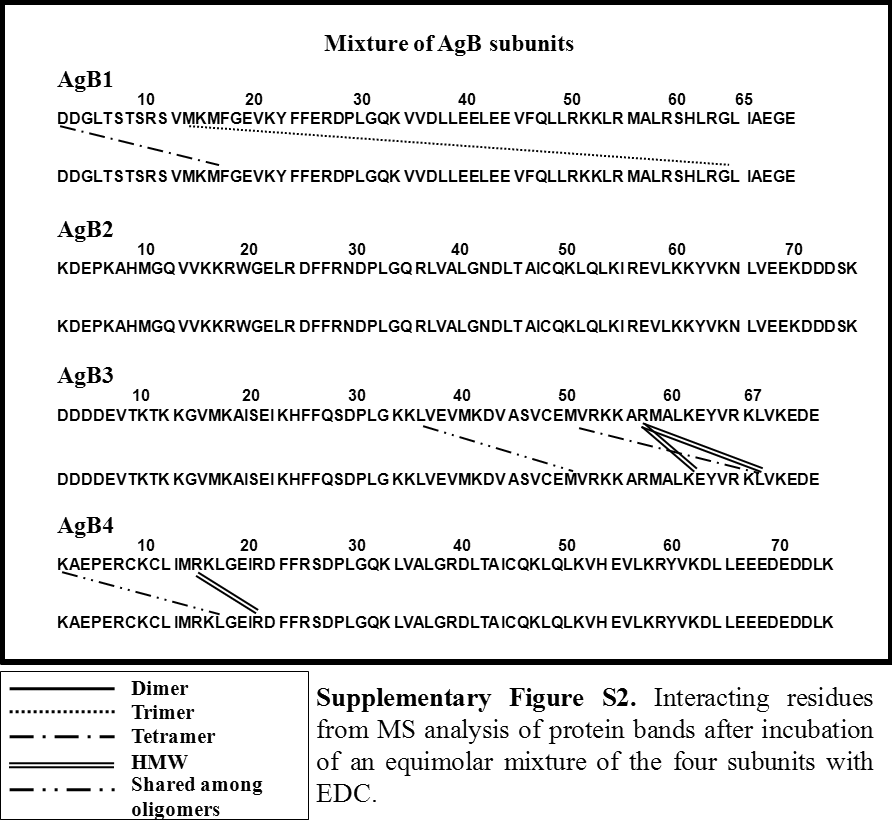

Supplement: Supplementary Figure S2 [file cix1006_suppl_supplementary_figure_s2.png]

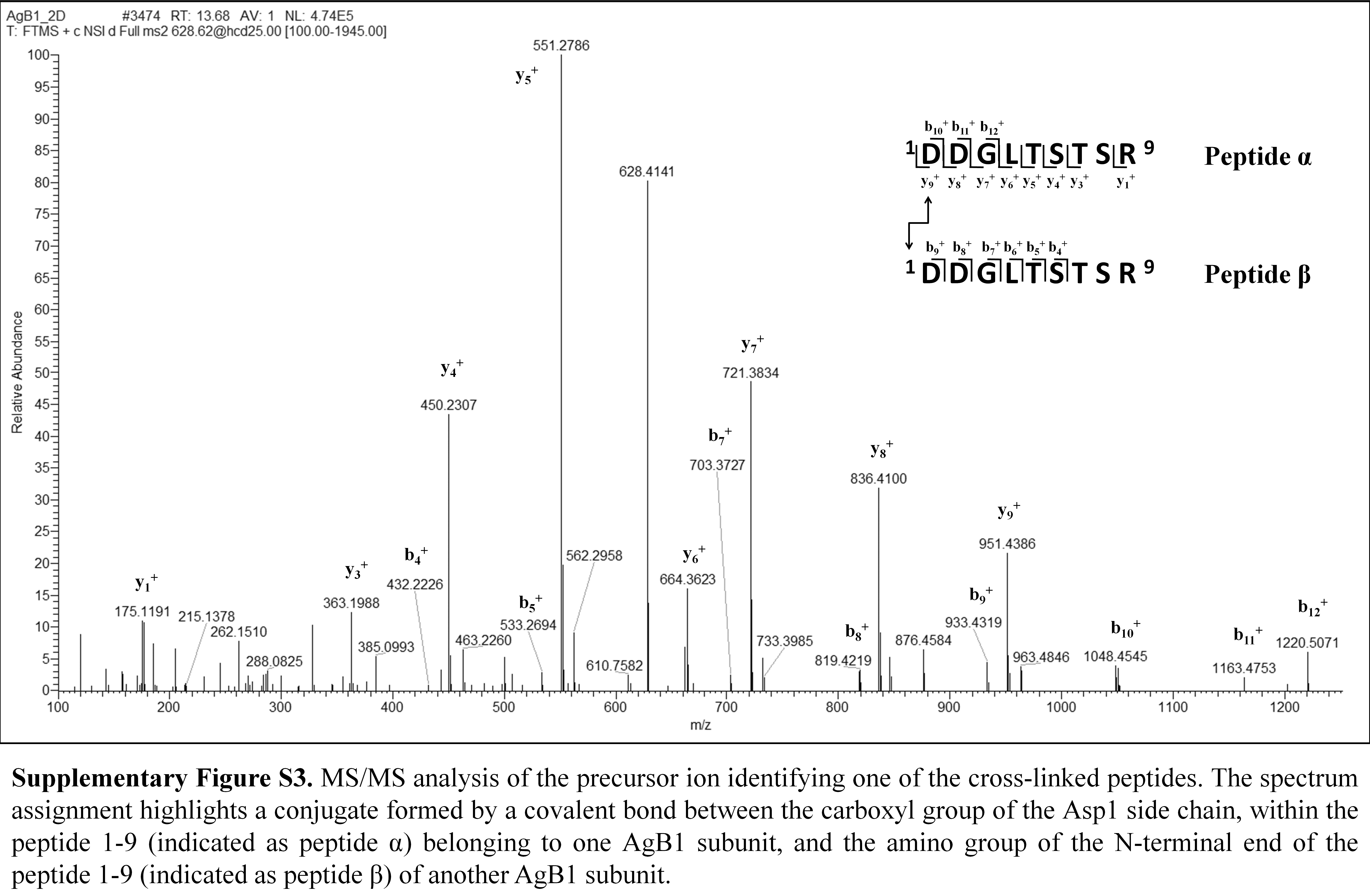

Supplement: Supplementary Figure S3 [file cix1006_suppl_supplementary_figure_s3.png]

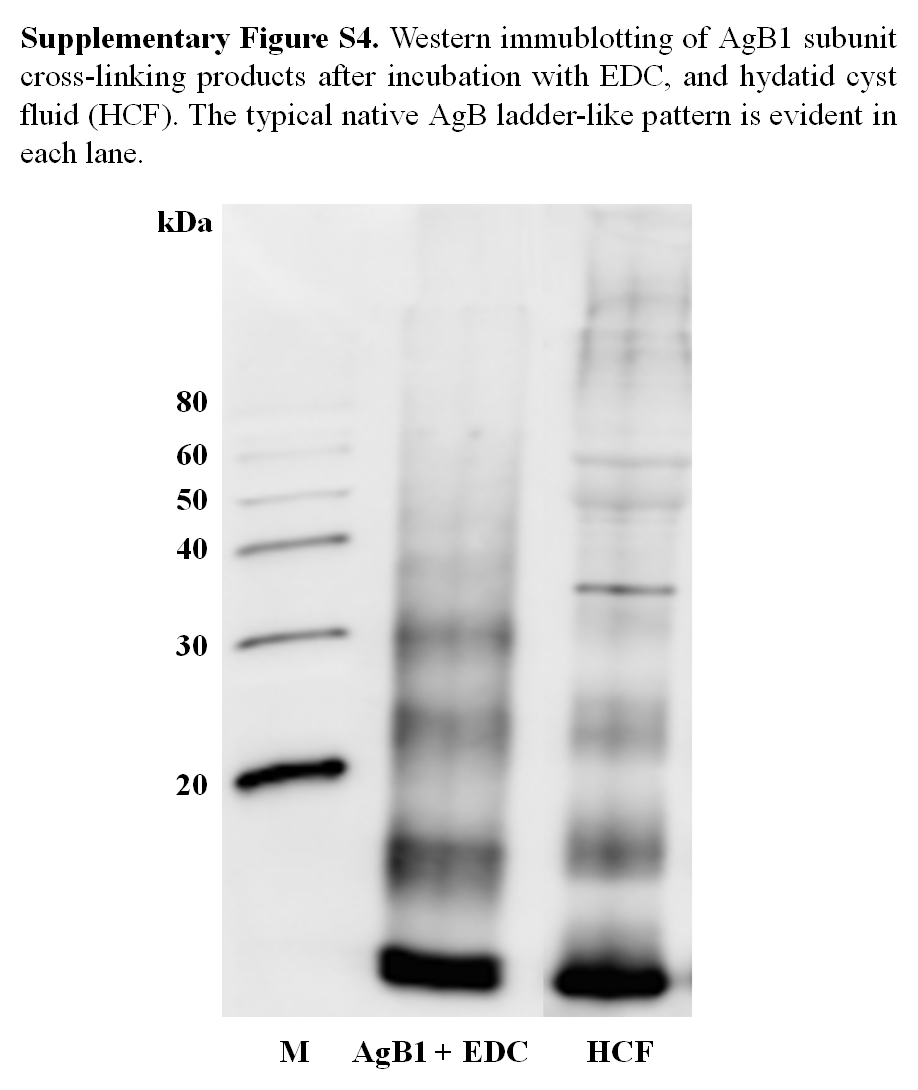

Supplement: Supplementary Figure S4 [file cix1006_suppl_supplementary_figure_s4.png]
