# Supplementary material for: Structural and Immunodiagnostic Characterization of Synthetic Antigen B Subunits From Echinococcus granulosus and Their Evaluation as Target Antigens for Cyst Viability Assessment
Source: Clin Infect Dis. 2017 Nov 15;66(9):1342–51. doi: 10.1093/cid/cix1006 (PMC5905600; doi:10.1093/cid/cix1006)
Supplement: Supplementary File S1 [file cix1006_suppl_supplementary_file_s1.docx]

**Methods**

**Synthesis and purification of AgB subunits**

Sequences of AgB1 (7.6 kDa), AgB2 (8.2 kDa), AgB3 (7.7 kDa) and AgB4 (8.2 kDa) were selected from UniProt/KB (D9J2I2_ECHGR, D9J2I3_ECHGR, D9J2I4_ECHGR and D9J2I5_ECHGR, respectively), synthesized, purified and verified by mass spectrometry (MS) as described previously [27, 28]. Briefly, proteins were synthesized by (Fmoc)-based solid-phase peptide synthesis on a Liberty (CEM Corporation Matthews, NC) peptide synthesizer, using a single-mode Discover SPS reactor. Products were purified by reverse phase-high pressure liquid chromatography (RP-HPLC), on a HP 1200 instrument (Agilent Technologies, Santa Clara, CA) equipped with binary pump, and UV-VIS detector. Runs were performed on a Jupiter-C18 column (250 mm × 10.00 mm, 5 μm, Phenomenex, Torrance, CA) using a 25 min gradient from 5 to 95% eluent B (0.07% trifluoroacetic acid in 95% acetonitrile) in eluent A (0.07% trifluoroacetic acid), at a flow rate of 4 mL/min. Elution profiles were recorded following the UV absorption at 220 nm. Purified proteins were then analyzed on a Q-TOF mass spectrometer equipped with a nano Z-spray source (Waters, Manchester, UK). Data were elaborated using the MassLynx program (Waters). Mass spectra of synthesized proteins confirmed the theoretical molecular weights.

**Size exclusion chromatography**

Size exclusion chromatography was performed by Fast Protein Liquid Chromatography (FPLC) on an AKTA Explorer 10 system (GE Healthcare, Uppsala, Sweden). Aliquots of 80 µg of each subunit were solubilized in 500 µL of 50 mM phosphate buffer, pH 7.4, containing 150 mM NaCl (running buffer), incubated at RT for 30 min, filtered at 12,000 *x g* on PVDF 0.22 µm filters (Ultrafree-MC Centrifugal Filter Units, Merck Millipore, Billerica, MA), and then loaded onto a Superdex 75 column (10/300 GL, GE Healthcare). Runs were performed at room temperature at a flow rate of 0.5 mL/min and elution profiles were recorded based on the UV absorption at 220 and 280 nm. Column was calibrated, under the same conditions, with appropriate standards (Aprotinin, Ribonuclease A, Carbonic Anhydrase, Ovalbumin, Conalbumin, and Blue Dextran 2000, Low Molecular Weight calibration kit, GE Healthcare). The obtained standard curve was used to evaluate the apparent molecular weight of AgB subunits by means of their experimental elution volumes.

**Chemical cross-linking experiments**

Cross-linking reactions were carried out in a total volume of 20 µL of 50 mM phosphate buffer, pH 7.4, 150 mM NaCl, to mimic the physiological conditions. Each AgB subunit (0.5 nmol) or their equimolar mixture (0.125 nmol/subunit) were incubated at 37°C for 15 minutes and then treated with a 100-fold molar excess of 1-ethyl-3-(3-dimethylamino propyl)-carbodiimide (EDC) cross-linking reagent for 30 minutes. Once demonstrated that the oligomers took place under these conditions, the method was optimized by incubating AgB subunits in 50 mM MES, pH 5.5, the buffer of choice for EDC . Reactions were stopped by quenching the excess of EDC with Laemmli buffer [29] with or without 400 mM dithiothreitol (DTT), as reducing agent. Protein samples were heated at 100 °C for 5 min and separated by SDS-PAGE on Any kD precast or 15% gels (Bio-Rad Laboratories, Hercules, CA). Proteins were stained by SimplyBlue SafeStain (Invitrogen, Carlsbad, CA), and digitalized with an ImageScanner III (GE Healthcare). Selected protein bands were excised from the gel, *in situ* digested with trypsin [30], and analyzed by MS for cross-linked regions identification. LC-MS/MS analyses were carried out using a Q Exactive mass spectrometer interfaced with an UltiMate 3000 RSLCnano LC system (Thermo Fisher Scientific, San Jose, CA). After loading, peptide mixtures were concentrated and desalted on a trapping pre-column (Acclaim PepMap C18, 75 μm × 2 cm nanoViper, 3 μm, 100 Å, Thermo Fisher Scientific), using 0.2% formic acid at a flow rate of 5 μl/min. The peptide separation was performed at 35 °C on a C18 column (EASY-Spray column, 15cm x 75μm ID, PepMap C18, 3μm, Thermo Fisher Scientific) at a flow rate of 300 nL/min, using a 25 min gradient from 1 to 50% eluent B (0.2% formic acid in 95% acetonitrile) in eluent A (0.2% formic acid in 5% acetonitrile). MS data were acquired using a data-dependent top12 method dynamically choosing the most abundant precursor ions from the survey scan, under direct control of the Xcalibur software (version 1.0.2.65 SP2, Thermo Fisher Scientific), where a full-scan spectrum (from 300 to 1,700 m/z) was followed by tandem mass spectra (MS/MS) of the 12 most abundant precursor ions. The instrument was operated in positive mode with a spray voltage of 1.8 kV and a capillary temperature of 275°C. Survey and MS/MS scans were performed with resolution of 70,000 and 17,500 at 200 m/z, respectively. The automatic gain control was set to 1,000,000 ions and the lock mass option was enabled on a protonated polydimethylcyclosiloxane background ion, as internal recalibration for accurate mass measurements [31]. The dynamic exclusion was set to 30 seconds. Higher Energy Collisional Dissociation (HCD), performed at the far side of the C-trap, was used as fragmentation method, by applying a 25eV value for normalized collision energy, and an isolation width of m/z 2.0. Nitrogen was used as the collision gas.

Cross-linked peptides were identified using StavroX software (version 3.3.0.1) [32], with the following settings: variable modification methionine oxidation, precursor precision 3 ppm, fragment ion precision 10 ppm, signal to noise ratio 2.0, and false discovery rate < 1%.

**Western immunoblotting**

Western immunoblotting was performed as described previously [25], with minor modifications. Briefly, a total of 200 ng of protein was loaded in each lane and separated by SDS-PAGE on 15% gels, electrically transferred onto nitrocellulose membranes and blocked in phosphate buffered saline, pH 7.4, 0.05% Tween 20 (PBS–T), containing 5% skim milk for 2 h. Membranes were then incubated for 1 h with Working Standard Anti-Echinococcus Serum, Human (WSH serum) (NIBSC, Potters Bar, UK), 1:200 in PBS-T, 2% skim milk; after washing, membranes were incubated with horseradish peroxidase conjugated anti-human IgG (Sigma-Aldrich, St. Louis, MO) diluted 1:500,000 in 2% skim milk in PBS-T, developed with ECL substrate (Sigma-Aldrich), and digitized with VERSA DOC 4000 MP (Bio-Rad).

**Samples**

Sera from patients with hepatic and extra-hepatic abdominal CE were collected at the Department of Infectious and Tropical Diseases of the IRCCS San Matteo Hospital Foundation, Pavia, Italy, WHO Collaborating Centre for Clinical Management of Cystic Echinococcosis, as part of routine management. CE cysts were classified by ultrasound according to the WHO-IWGE classification.

Sera from healthy subjects, collected at the Hospital Blood Donor Center of Sassari, Italy, were used as a control group. A written informed consent to the use of leftover anonymized sera for research purposes was obtained at the time of sample collection. The study was approved by the ethics committees of IRCCS San Matteo Hospital Foundation, Pavia, Italy, Prot N. 20150004877, for sera from patients with CE, and by the local health authority of Sassari (ASL N. 1, Sassari), Prot N. 1123/L, for sera from blood donors.

A total of 422 blood sera were available, including 148 from patients with CE cysts, 25 from patients that had previously been treated surgically for CE and in follow-up at the time of serum collection, and 249 from healthy controls. Table 1 summarizes the number of patients available for each group and the corresponding cyst location. Single cysts were found in 78 CE patients (52.7%), whilst multiple cysts were found in the other 70 CE patients (47.3%). When more than one CE cyst was present in a single patient, the serum was classified according to the cyst stage known to have the higher odds of resulting in a positive serology: CE2-CE3b-CE3a>CE1>CE4-CE5 [3], irrespective of the location of this cyst.

**ELISA**

ELISA was performed as described previously [26]. Microplates (Nunc-Maxisorp Immunoplate, Waltham, MA) were coated with 100 µL/well of differently concentrated antigen solutions (250 ng/mL, 330 ng/mL, 500 ng/mL, 1000 ng/mL, respectively) in phosphate buffered saline (PBS). After blocking and washings, sera were added at 1:200 dilutions in 2% bovine serum albumin in PBS-0.05% tween-20 (BSA in PBS-0.05%T) and incubated at 37 °C for 1 hour. Horseradish peroxidase conjugated anti-human IgG (Sigma-Aldrich) was diluted 1:100,000 in 2% BSA in PBS-0.05%T and incubated at 37° C for 1 hour. Finally, the substrate (3,3',5,5'-Tetramethylbenzidine Liquid Substrate, Supersensitive, Sigma-Aldrich) was added. The absorbance was read at 620 nm after 1 hour incubation using a Tecan Sunrise (Tecan Group Ltd., Männedorf, Switzerland) microplate reader. All sera were tested in duplicate.

In order to compare results obtained from different plates, a Sample Ratio (SR) was calculated as reported previously [26].

**Statistical analysis**

Data analysis was performed with MedCalc Statistical Software version 15.2.2 (MedCalc Software bvba, Ostend, Belgium; http://www.medcalc.org; 2015). A receiver-operator characteristic analysis (ROC) was performed to determine a cut-off value for each AgB based test. The standard error and the area under the curve were calculated according to DeLong et al. [33]. Levels of sensitivity were plotted against levels of 100 minus specificity at each cut-off point on a ROC curve. Threshold values used were those associated with the highest Youden index J [34]. In order to calculate the best ELISA cut-off values, and to improve sensitivity on active-transitional cysts, ROC curves were built by using SR values from patients with CE1, CE2, CE3a and CE3b as positive group (94 sera) and healthy controls as negative group (249 sera). The area under the ROC curve (AUC) was used to define the antigen discriminatory power (between subjects with active-transitional cysts and subjects with inactive cysts or without the disease). A *p*-value <0.05 was considered statistically significant. McNemar test was performed, on all 422 sera (patients, post surgery and healthy), to compare the sensitivities of the two AgB ELISAs. Differences in SR values between groups were analyzed by Kruskal-Wallis test; when more than two groups were analyzed, after Kruskal-Wallis test, pairwise multiple comparisons were evaluated by Conover test with Bonferroni correction [35, 36].
